# SUMO Pathway Dependent Recruitment of Cellular Repressors to Herpes Simplex Virus Type 1 Genomes

**DOI:** 10.1371/journal.ppat.1002123

**Published:** 2011-07-14

**Authors:** Delphine Cuchet-Lourenço, Chris Boutell, Vera Lukashchuk, Kyle Grant, Amanda Sykes, Jill Murray, Anne Orr, Roger D. Everett

**Affiliations:** MRC-University of Glasgow Centre for Virus Research, Glasgow, Scotland, United Kingdom; McMaster University, Canada

## Abstract

Components of promyelocytic leukaemia (PML) nuclear bodies (ND10) are recruited to sites associated with herpes simplex virus type 1 (HSV-1) genomes soon after they enter the nucleus. This cellular response is linked to intrinsic antiviral resistance and is counteracted by viral regulatory protein ICP0. We report that the SUMO interaction motifs of PML, Sp100 and hDaxx are required for recruitment of these repressive proteins to HSV-1 induced foci, which also contain SUMO conjugates and PIAS2β, a SUMO E3 ligase. SUMO modification of PML and elements of its tripartite motif (TRIM) are also required for recruitment in cells lacking endogenous PML. Mutants of PML isoform I and hDaxx that are not recruited to virus induced foci are unable to reproduce the repression of ICP0 null mutant HSV-1 infection mediated by their wild type counterparts. We conclude that recruitment of ND10 components to sites associated with HSV-1 genomes reflects a cellular defence against invading pathogen DNA that is regulated through the SUMO modification pathway.

## Author Summary

Viruses encounter several different defences that impede infection, including acquired immunity mediated by the immune system and innate immunity that includes the synthesis of antiviral proteins through the interferon pathway. In recent years, a third arm of antiviral defence has been described, named intrinsic immunity or intrinsic resistance, that is conferred by constitutively expressed cellular proteins. In the case of herpesviruses, intrinsic resistance involves the action of cellular repressors that restrict viral transcription once the viral genome enters the nucleus. Several studies have presented evidence that one aspect of intrinsic resistance involves cellular proteins that form distinct nuclear structures known as ND10. Several ND10 components are known to accumulate rapidly at sites in close association with herpes simplex virus type 1 genomes. Here we report that this cellular response requires the ability of several of the proteins in question to interact with a small ubiquitin-like protein known as SUMO. In two such examples of these proteins, we show that their ability to interact with SUMO is required for their roles in repressing viral infection. We suggest that this SUMO-dependent pathway may underlie a more general mechanism by which cells protect themselves from invading foreign DNA.

## Introduction

Herpesvirus infections are controlled by acquired and innate defences involving cellular, humoral and cytokine mediated responses (for reviews, see [1]). In recent years a concept has emerged of an additional antiviral defence mechanism that operates within individual cells. Unlike cytokine-mediated responses, intrinsic antiviral resistance involves the actions of pre-existing cellular proteins that, in the case of herpesviruses, act to repress viral transcription [2], [3], [4]. This defence is counteracted by viral regulatory proteins, for example the immediate-early (IE) proteins ICP0 of herpes simplex virus type 1 (HSV-1) [5], [6], [7], ie1 (IE72) of human cytomegalovirus (HCMV) [8], and HCMV virion component pp71 [9], [10], [11], [12], [13], [14]. One aspect of intrinsic resistance concerns cellular nuclear sub-structures known as ND10 or promyelocytic leukaemia (PML) nuclear bodies, and a number of their major components, namely PML itself, Sp100, hDaxx and ATRX. In HSV-1 infections, ICP0 overcomes the repressive properties of these proteins by inducing their degradation or dispersal [7], [15], [16], [17]. ICP0 null mutant HSV-1 exhibits a greatly reduced plaque forming efficiency, but this defect is partially reversed in cells depleted of PML, Sp100, hDaxx or ATRX [5], [6], [7].

A notable feature of PML and other ND10 components is their recruitment to novel ND10-like foci that are closely associated with parental HSV-1 genomes and early replication compartments during the initial stages of infection [18], [19]. The recruitment of PML to the virus-induced foci is not dependent on *de novo* viral protein expression and occurs extremely rapidly, indicating that the cell responds to the entry of viral genomes into the nucleus [18], [20]. Although the effect can be seen in wild type (wt) HSV-1 infections, it is short lived as the recruited proteins are rapidly degraded or dispersed through the effects of ICP0. During infection with ICP0 null mutant HSV-1, however, the ND10 proteins remain in these novel sites in a much longer-lived manner. The correlation between the biological activity of many ICP0 mutant proteins and their ability to counteract this recruitment process [21] suggests that this phenomenon reflects an aspect of intrinsic antiviral resistance. This model proposes that the recruited proteins generate a repressive environment that impedes viral transcription. This paper concerns the molecular mechanism by which ND10 components are recruited to the virus-induced foci and tests the hypothesis that the recruitment process contributes to intrinsic resistance to HSV-1 infection.

The formation of the virus-induced ND10-like structures is distinct from that of normal ND10 in uninfected cells because it is not dependent on PML or indeed any of the major ND10 components that have so far been studied [5], [6], [7], [20]. Here, we have used a depletion/reconstitution approach to analyze the molecular requirement for the recruitment of PML, Sp100 and hDaxx to HSV-1 genome-associated sites in newly infected cells. We found that in all cases the presence of a SUMO interaction motif (SIM) [22] is required for this property, and that the major SUMO modification sites of PML, but not Sp100, are also required. The virus-induced ND10-like foci also contain SUMO-2/3 conjugates and PIAS2β, a SUMO E3 ligase, even in the absence of PML. Unlike their wt counterparts, SIM deletion mutants of PML isoform I and hDaxx are unable to repress ICP0 null mutant HSV-1 infection. These data demonstrate that SUMO modification pathways play a key role in the recruitment of ND10 proteins to HSV-1 genome associated sites. We propose that this SUMO-dependent cellular response is an important component of intrinsic cellular defence against foreign DNA that has entered the nucleus.

## Results

### Recruitment of PML isoforms to sites associated with HSV-1 genomes

**Figure 1:**
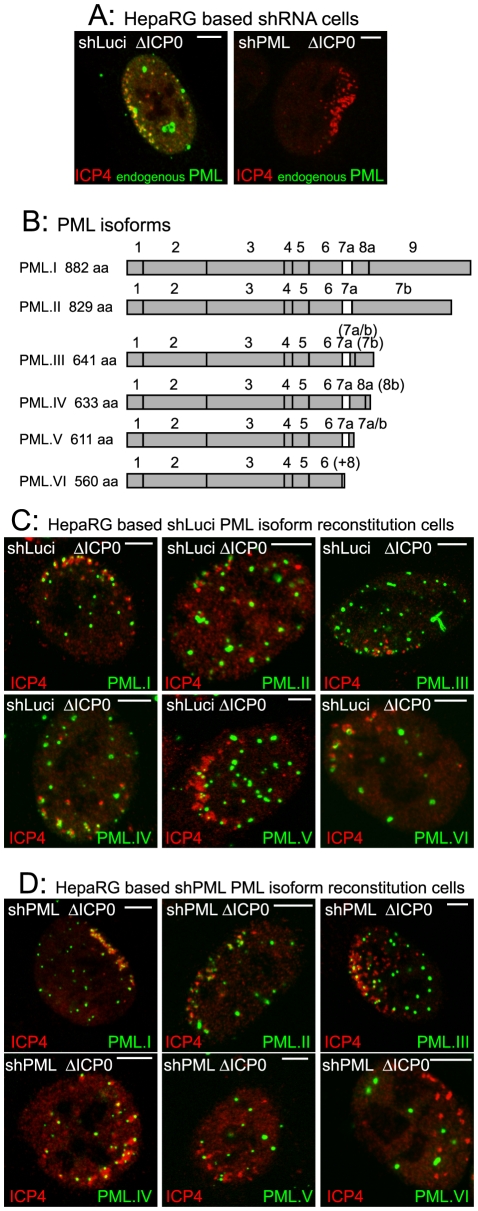
The major nuclear isoforms of PML and their recruitment to sites associated with HSV-1 genomes. A. Typical examples of recruitment of endogenous PML (green) to sites associated with HSV-1 genomes (ICP4: red) in cells at the edges of ICP0 null mutant (ΔICP0) plaques in control and PML depleted cells. B. PML isoforms I to VI, noting the included exons and the translated product length. The bracketed exons indicate the use of alternative reading frames for these sequences. (+8) at the end of PML VI indicates an additional 8 residues following exon 6 as a result of alternative splicing that deletes exon 7a. Adapted from [23]. C and D. Recruitment of EYFP-PML isoforms in control (panel C) and PML depleted cells (panel D). Scale bars indicate 5 µm. Analysis of the localization of these proteins in uninfected cells and their expression levels is presented elsewhere [23].

The recruitment of PML and other proteins to sites associated with parental HSV-1 genomes and early replication compartments can be detected by examination of cells at the periphery of developing viral plaques. The viral genomes enter the nucleus of newly infected cells in a directional and asymmetric manner, and remain close to the nuclear envelope. The viral IE transcriptional regulator ICP4 avidly binds to viral DNA, and therefore forms foci that, in cells at the early stages of infection, are commonly distributed along one interior edge of the nucleus. PML and other ND10 proteins then accumulate at novel sites that are closely associated with the viral genomes [18], [19]. This phenotype allows the unambiguous identification of proteins that have been recruited to and accumulate at the virus-induced sites. A typical example of endogenous PML recruitment in an ICP0-null mutant HSV-1 (ΔICP0) infected HepaRG cell expressing a control shRNA is shown in Figure 1A (left) in comparison with a similarly infected PML depleted cell (Figure 1A, right).

We investigated the molecular characteristics of PML that are required for this recruitment by adopting a depletion/reconstitution approach [23]. All the PML isoforms studied (Figure 1B), expressed as EYFP fusions, were recruited to sites associated with ICP4 foci in cells containing endogenous PML (Figure 1C). In cells depleted of endogenous PML, however, while PML isoforms I to V were recruited, PML.VI remained entirely in large foci that were not associated with ICP4 (Figure 1D). The separated channels of the images in Figures 1C and 1D are shown in Figures S1 and S2. This assay is not amenable to precise quantification as the extent of recruitment varies between cells and is most obvious when the ICP4 foci are small. Nonetheless, when recruitment occurs it is evident in all cells with ICP4 foci near the nuclear periphery. A large number of cells were examined in each experiment, and we have scored a protein as defective in recruitment only when its behaviour was clearly different from the appropriate control. As an example, compare PML.VI in the control and PML depleted backgrounds in Figures 1C and D. Although recruitment of PML proteins that we have scored as recruitment positive occurred to some extent in all relevant cells, we noted some differences in PML isoform behaviour. For example, PML.I was recruited more extensively than PML.V (Figure 1D). We have not investigated the basis of these differences.

The defect in PML.VI recruitment to virus-induced foci also occurred in analogous human fibroblast (HF) derived cells. PML.I co-localized with Sp100 in both control and PML depleted HFs (Figure S3), both PML.I and PML.VI were recruited to the virus-induced in control HFs (Figure S4A), but PML.VI was not recruited in PML depleted HFs (Figure S4B).

PML isoform VI includes all the conserved exons present in the other isoforms, except exon 7a (Figure 1B). Therefore exon 7a includes sequences that are required for recruitment of PML to the virally induced foci. The defect in PML.VI recruitment is not exhibited when endogenous PML is present because PML interactions mediated through the coiled-coil motif [24], [25] enable the defective protein to be recruited in partnership with the endogenous isoforms.

### PML exon 7a is required for recruitment to sites associated with HSV-1 genomes

**Figure 2 ppat-1002123-g002:**
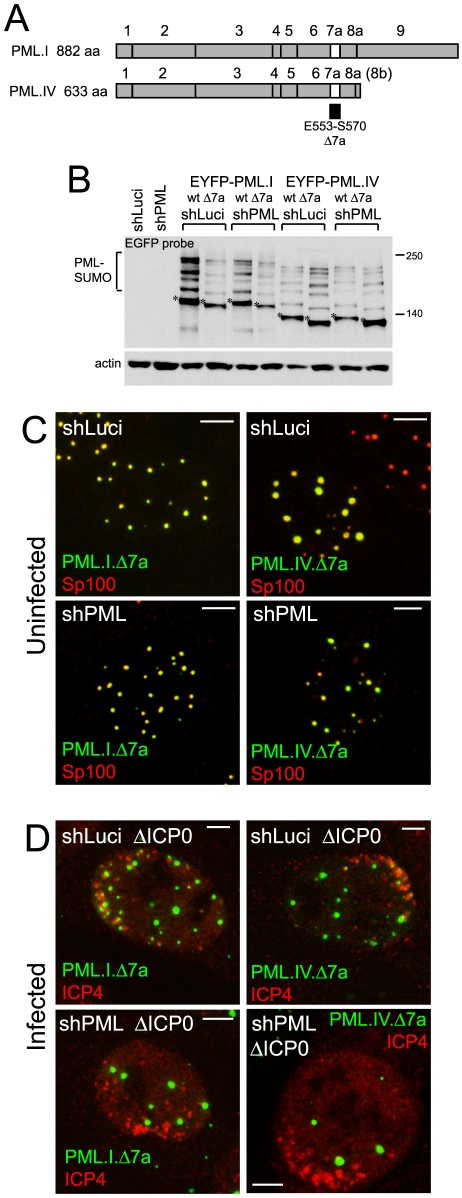
SIM deletion mutants of PML are defective in recruitment to sites associated with HSV-1 genomes. A. Maps of PML isoforms I and IV and derivatives lacking exon 7a. B. Western blot of the EYFP-linked proteins in control and PML depleted cells using an anti-EGFP antibody. The major unmodified bands of the EYFP-PML proteins are indicated by asterisks. C. Localization of EYFP-PML proteins (green) and endogenous Sp100 (red) in uninfected control and PML depleted cells. D. Typical assays of recruitment of EYFP-proteins (green) to sites associated with HSV-1 genomes (ICP4; red) in ICP0 null mutant (ΔICP0) infected control and PML depleted cells. Scale bars indicate 5 µm.

PML exon 7a encodes an 18 amino acid segment containing a SIM that has been implicated in proper ND10 assembly [26]. Mutants of PML.I and PML.IV that lack exon 7a (Figure 2A). were expressed at levels similar to their wt versions, with similar patterns of SUMO modification, and co-localized with Sp100 in both control and PML depleted cells (Figure 2B and C). While both mutants were recruited to virus-induced foci in the presence of endogenous PML, both were defective in recruitment in PML depleted cells (Figures 2D and S5A). This defect of PML.I.Δ7a was confirmed in HF derived cells (Figure S5B and C; the localization of this protein in uninfected HFs is shown in Figure S3). EYFP-PML.I with substitution mutations in the SIM (PML.I.mSIM) exhibited properties very similar to those of PML.I.Δ7a in uninfected and ICP0-null mutant HSV-1 infected control and PML depleted cells (Figure S6). These data confirm that the lack of recruitment of PML.VI is due to the absence of the SIM in exon 7a.

### The role of SUMO modification in the recruitment of PML to virus-induced foci

**Figure 3:**
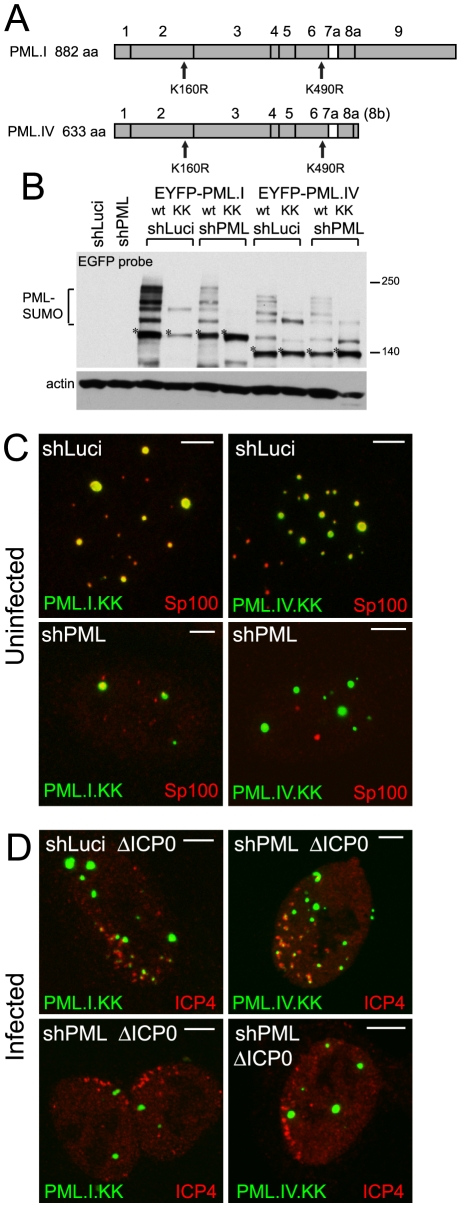
SUMO modification mutants of PML are defective in recruitment to sites associated with HSV-1 genomes. A. Maps of PML isoforms I and IV and derivatives with lysine to arginine substitutions at residues K160 and K490. B. Western blot of these proteins in control and PML depleted cells, using an anti-EGFP antibody. The major unmodified bands of the EYFP-PML proteins are indicated by asterisks. C. Localization of the EYFP-PML proteins (green) and endogenous Sp100 (red) in uninfected control and PML depleted cells. D. Typical assays of recruitment of these PML proteins (green) to sites associated with HSV-1 genomes (ICP4; red) in ICP0 null mutant (ΔICP0) infected control and PML depleted cells. Scale bars indicate 5 µm.

SUMO modification of PML is essential for ND10 assembly in uninfected cells [27]. Therefore we investigated whether this modification, in addition to the SIM, plays a role in PML recruitment to the virus-induced foci. Mutants of PML.I or PML.IV with lysine to arginine substitutions at residues 160 and 490 (PML.I.KK and PML.IV.KK; Figure 3A) exhibit substantial defects in SUMO modification, but some apparently modified species remain, especially in cells containing endogenous PML, and more so with PML.IV than PML.I. Modification of PML.I.KK in PML depleted cells was almost completely eliminated (Figure 3B).

PML.I.KK and PML.IV.KK localized to ND10 normally in cells expressing endogenous PML, probably through interactions with endogenous PML via their coiled-coil domains (Figure 3C, upper row). In contrast, and consistent with previous work [27], PML.I.KK exhibited a drastically altered localization in PML depleted cells, forming a reduced number of foci of increased size, some of which were in the cytoplasm. The nuclear foci contained greatly reduced amounts of Sp100. The aberrant PML.IV.KK foci were predominantly nuclear, again with little co-localization with Sp100 (Figure 3C, lower row). Residual co-localization with Sp100 of these PML mutants may be influenced by potential SUMO modification at other lysine residues. However, it is also affected by cell type since in the corresponding PML depleted HFs co-localization of PML.I.KK with Sp100 was more marked (Figure S9A).

To eliminate any residual SUMO modification of PML.I.KK we introduced additional mutations at either lysine 65 [28] or at lysine 616, which lies in a good match (LKID) to the consensus SUMO modification site (ΨKXE). Mutant PML.I.K123 (K65R, K160R, K490R) exhibited similar residual SUMO modification to that of PML.I.KK in cells expressing endogenous PML, whereas the K616R mutation virtually eliminated all modified bands in mutants PML.I.K234 (K160R, K490R, K616R) and PML.I.4KR (all indicated lysine residues substituted with arginine) (Figure S7). We conclude that PML.I is not detectably modified at lysine 65, but that lysine 616 is likely to be a SUMO modification site that is specific for PML.I and PML.IV (because it lies in exon 8a which is present in these two isoforms only). We note that lysine 65 is very close to zinc coordinating cysteine residues in the RING finger, so SUMO modification here would likely affect both the overall architecture of the RING and its interaction interfaces. PML.I.4KR co-localized with Sp100 in control but not PML depleted cells, in both HepaRG and HF backgrounds (Figures S8A and S9A). These data support the hypothesis that the sporadic co-localization of PML.I.KK with Sp100 in PML depleted cells (Figure 3C) is due to the remaining potential for SUMO modification.

As in the case of the SIM mutants, both PML.I.KK and PML.IV.KK could be recruited to virus-induced foci in cells expressing endogenous PML (Figure 3D, upper row), but recruitment was either absent or at greatly reduced levels in PML depleted cells (Figure 3D, lower row, and Figure S8B). Similar results were obtained in the equivalent HF derived cells (Figure S9B), and with the 4KR mutant in both HepaRG and HF backgrounds (Figures S8C and S9B). Therefore, in addition to the role of the SIM, mutation of the major SUMO modification sites of PML severely impedes the capacity of the protein to be recruited to sites associated with viral genomes, providing endogenous PML isoforms are absent.

### Absence of the SIM does not affect the intrinsic mobility of PML

**Figure 4:**
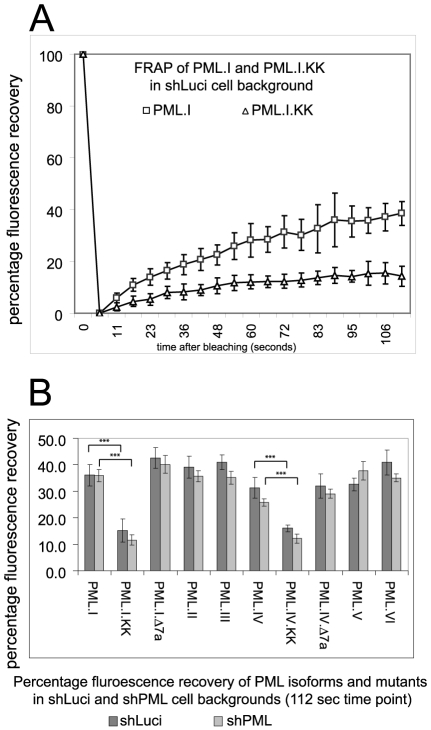
FRAP analysis of wt PML isoforms and SUMO modification and SIM deletion mutants of PML.I and PML.IV in control and PML depleted cells. A. Examples of the original data of PML.I and PML.I.KK in control HepaRG shLuci cells.B. The means and standard deviations of the percentage recoveries at the final time point for each of the proteins in both cell backgrounds. Statistical significance between the indicated data sets was calculated by Student's two-tailed t-Test, p = <0.0001.

The defect of mutant PML proteins in recruitment to virus-induced foci could be explained by a decrease in intrinsic mobility. The dynamics of PML isoforms and SUMO modification deficient mutants in both the presence and absence of endogenous PML have been reported [18], [29], [30], [31], but the role of the SIM has not been investigated before. We analyzed the dynamics of all EYFP-linked PML isoforms, and the Δ7a and KK mutants of PML.I and PML.IV, in both control and PML depleted HepaRG cells. Typical FRAP recovery plots of PML.I and PML.I.KK in control cells are presented in Figure 4A, confirming previous conclusions that SUMO modification is required for the normal mobility of PML [30], [32]. The data for all PML isoforms and mutants in both cell backgrounds are presented as their degree of fluorescence recovery at the 112-second time point (Figure 4B). The results are broadly in agreement with previous work, with the exception that the relatively slow recovery of PML.V [30] was not reproduced here. The only major differences were between the SUMO modification deficient mutants and the wt forms. Importantly, the defect in recruitment to virus-induced foci of PML.VI and the PML.I and PML.IV mutants lacking exon 7a cannot be explained simply by reduced mobility, whereas in the case of the SUMO modification mutants this explanation cannot be excluded.

### The role of elements within the TRIM in recruitment of PML to virus-induced foci

**Figure 5:**
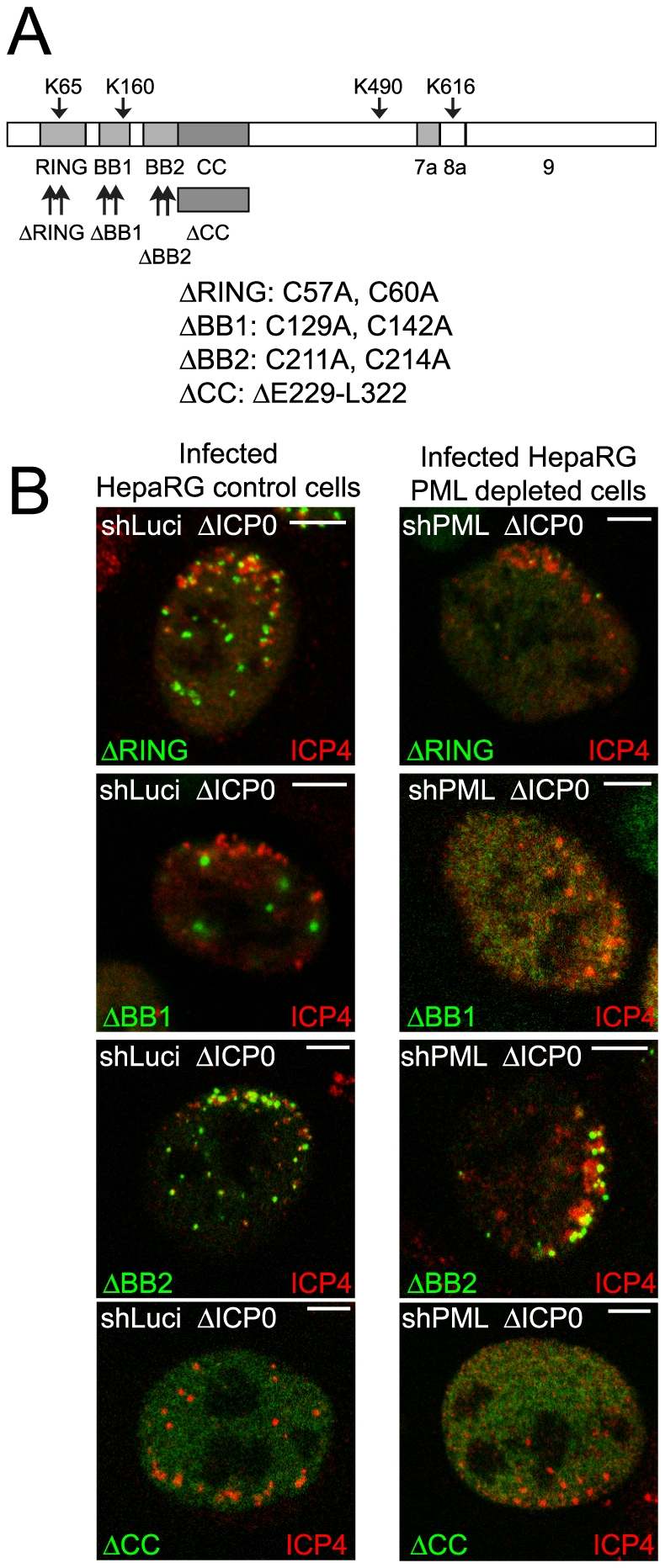
Most TRIM mutants of PML are defective in recruitment to sites associated with HSV-1 genomes. A. Map of the conserved PML.I showing the TRIM elements and the mutations introduced into EYFP-PML.I, adapted from [23]. Analysis of the localization of these proteins in uninfected cells and their expression levels is presented elsewhere [23]. B. Typical assays of recruitment of these PML proteins (green) to sites associated with HSV-1 genomes (ICP4; red) in cells at the edges of ICP0 null mutant (ΔICP0) plaques in control and PML depleted cells (left- and right-hand sets of images, respectively). Scale bars indicate 5 µm.

Control and PML depleted cell lines that express EYFP-PML.I with a deletion of the coiled-coil or point mutations in the RING finger, B-Box 1 or B-Box 2 (Figure 5A) have been described previously [23]. Briefly, the RING finger mutant (ΔRING) co-localized with endogenous ND10 in control cells but was mostly nuclear diffuse in cells lacking endogenous PML, the coiled-coil deletion mutant (ΔCC) was nuclear diffuse in both cell backgrounds, the B-Box 2 mutant (ΔBB2) formed foci in both types of cell but in neither case were these co-localized with Sp100, and the B-Box 1 mutant (ΔBB1) co-localized with Sp100 in aberrant ND10-like structures in control cells and formed foci in a proportion of PML depleted cells, none of which co-localized with Sp100. SUMO modification of all tripartite motif (TRIM) mutants was highly compromised in both cell backgrounds [23].

The ΔRING and ΔBB2 mutants were recruited efficiently to the virus-induced foci in cells expressing endogenous PML, whereas the ΔBB1 and ΔCC mutants were not (Figures 5B and S10A). The ΔBB2 mutant was also efficiently recruited in PML depleted cells (Figure 5B), even though this mutant was not well SUMO-modified and did not efficiently co-localize with Sp100 in uninfected cells in either cell background [23]. Therefore the characteristics of PML required for nucleating an ND10-like structure in uninfected cells are distinguishable from those involved in recruitment to the novel virus-induced structures. As in cells expressing endogenous PML, the ΔBB1 and ΔCC mutants were not recruited into virus-induced foci in PML depleted cells (Figure 5B). The most variable results were obtained with the ΔRING mutant in PML depleted cells. In most cells infected with ICP0 null mutant HSV-1 this mutant remained nuclear diffuse, but in some cells it was recruited with variable efficiencies (Figure 5B). We conclude that whereas B-Box 1 and the coiled-coil are essential for PML recruitment to virus induced foci, B-Box 2 is dispensable, and inactivation of the RING greatly diminishes but does not always eliminate recruitment.

### The SIM of Sp100 is required for recruitment to HSV-1 induced foci

**Figure 6:**
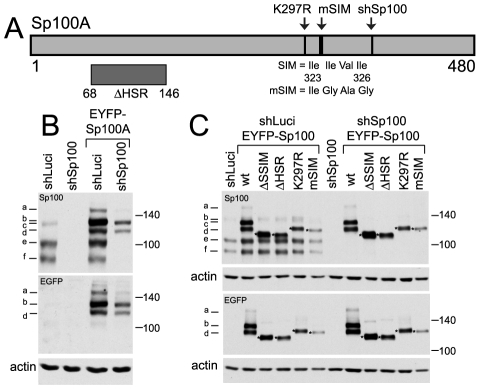
Expression and analysis of mutants of Sp100A and their recruitment to sites associated with HSV-1 genomes. A. Map of Sp100A and mutations introduced into EYFP-Sp100A fusion proteins. The sequence targeted by the anti-Sp100 shRNA and the silent mutations that render the mRNA resistant is marked (shSp100). B. Western blot of wt EYFP-Sp100A in control and Sp100 depleted cells, with anti-Sp100 and anti-EGFP antibodies (upper and lower parts, respectively). The bands are as follows: a - double modification of EYFP-Sp100A; b - single SUMO modification of EYFP-Sp100A; c - double modification of endogenous Sp100; d - unmodified EYFP-Sp100A; e - single SUMO modification of endogenous Sp100A; f - unmodified endogenous Sp100A. C. Western blot of wt and mutant Sp100 proteins in control and Sp100 depleted cells. The mutant ΔSSIM deletes residues 289–327 encompassing both the K297 SUMO modification site and the SIM.

**Figure 7:**
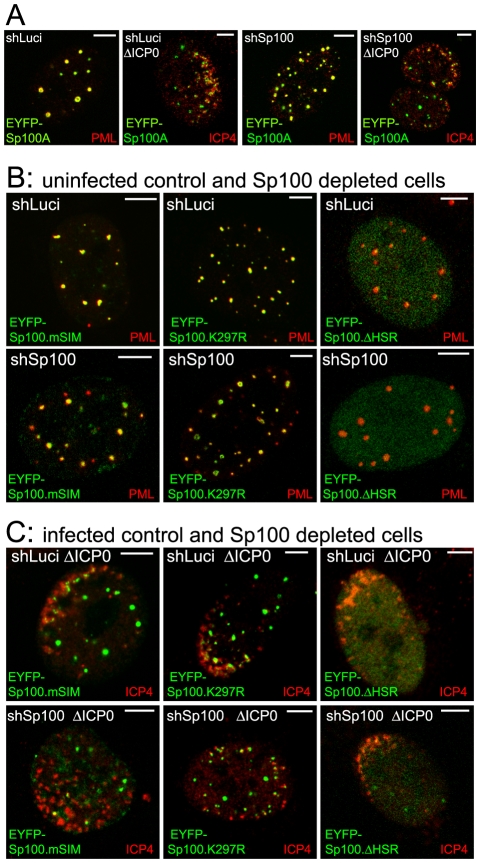
Immunofluorescence analysis of Sp100 reconstructed cells. A. Typical images of wt EYFP-Sp100A (green) in uninfected control and Sp100 depleted cells co-stained for endogenous PML (red), and its recruitment to sites associated with HSV-1 genomes (ICP4; red) in cells at the edges of ICP0 null mutant (ΔICP0) plaques. B. Typical images of the mSIM, K297R and ΔHSR mutant proteins (green) in uninfected control and Sp100 depleted cells co-stained for endogenous PML (red). C. Assays of recruitment of these mutant proteins to sites associated with HSV-1 genomes (ICP4; red) in cells at the edges of ICP0 null mutant (ΔICP0) plaques. Scale bars indicate 5 µm.

Given that recruitment of PML to sites to HSV-1 induced foci is dependent on its SIM, we investigated whether recruitment of other ND10 proteins is also SIM dependent. Sp100A is the smallest and most abundantly expressed Sp100 isoform. It includes a SIM (residues 323–326), a major SUMO modification site at lysine 297, and a region near the N-terminus that is required for localization to ND10 the HSR domain (Figure 6A) [33], [34]. Control and Sp100-depleted HepaRG cells [6] were transduced with lentivirus vectors expressing EYFP-linked Sp100A and mutants lacking the HSR domain (ΔHSR), the SUMO modification site (K297R) or the SIM (mSIM), or a combination of both K297R and mSIM mutations (ΔSSIM). Consistent with previous studies [33], [34], none of the mutant proteins exhibited the SUMO modification pattern characteristic of wt Sp100A (Figure 6C). The mSIM mutant of Sp100A at least partially co-localized with PML in control cells, and also in Sp100 depleted cells expressing higher levels of the recombinant protein (Figure 7B, leftmost panels), although it was nuclear diffuse when weakly expressed in Sp100 depleted cells (not shown).

Both wt and K297R mutant EYFP-Sp100A were recruited to the virus-induced foci in both control and Sp100 depleted cells (Figure 7A and C), indicating that, unlike PML, the major SUMO modification site is not required for Sp100A recruitment. Although the HSR deletion mutant was not so recruited (Figure 7C, right-most panels, and Figure S10B), it is possible that this deletion causes major structural defects because this mutant does not localize to ND10 in uninfected cells (Figure 7B). The Sp100A.mSIM and ΔSSIM mutants were recruited to virus-induced foci in cells containing endogenous Sp100, but not in its absence (Figure 7C, leftmost panels, and Figure S10B; data not shown for ΔSSIM). These results indicate that the SIM of Sp100, like that of PML, is required for recruitment, but in its absence the mSIM mutant can be recruited through an interaction with endogenous Sp100, most likely through the HSR domain [34].

### The SIM of hDaxx is required for its recruitment to HSV-1 induced foci

**Figure 8:**
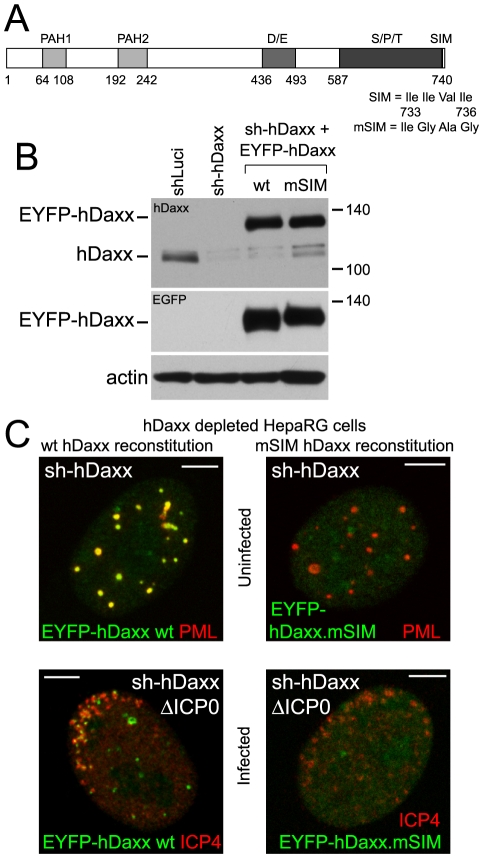
Recruitment of hDaxx to sites associated with HSV-1 genomes is dependent on its SIM. A. Map of hDaxx and its major features (PAH1 and PAH2 – paired amphipathic helices 1 and 2; D/E – aspartic and glutamic acid rich region; S/P/T – serine, proline and threonine rich region). The position and sequence of the SIM and the changes in the mSIM mutant are indicated. B. Western blot of control and hDaxx depleted cells, and depleted cells reconstituted with the wt and mSIM mutant EYFP-hDaxx, using anti-hDaxx (upper panel) and anti-EGFP (lower panel) antibodies. C. Typical images of wt and mSIM mutant EYFP-hDaxx (green) in uninfected hDaxx depleted cells co-stained for endogenous PML (red) (upper panels), and assays of their recruitment to sites associated with HSV-1 genomes (ICP4; red) in cells at the edges of ICP0 null mutant (ΔICP0) plaques (lower panels). Scale bars indicate 5 µm.

Like PML and Sp100, hDaxx includes a SIM (at its C-terminus; Figure 8A) and is also recruited to HSV-1 induced ND10-like foci [18], [35]. Both wt and mSIM mutant versions of hDaxx were expressed in control (data not shown) and hDaxx depleted cells (Figure 8B). The wt protein co-localized with PML in ND10 (Figure 8C, upper row, left), but the mSIM mutant was diffusely distributed in the nucleus (Figure 8C, upper row, right). As with PML and Sp100, wt but not SIM mutant EYFP-hDaxx was recruited to the virus-induced foci in hDaxx depleted cells (Figures 8C and S10C).

### PIAS2β and SUMO isoforms are present at HSV-1 induced foci

**Figure 9:**
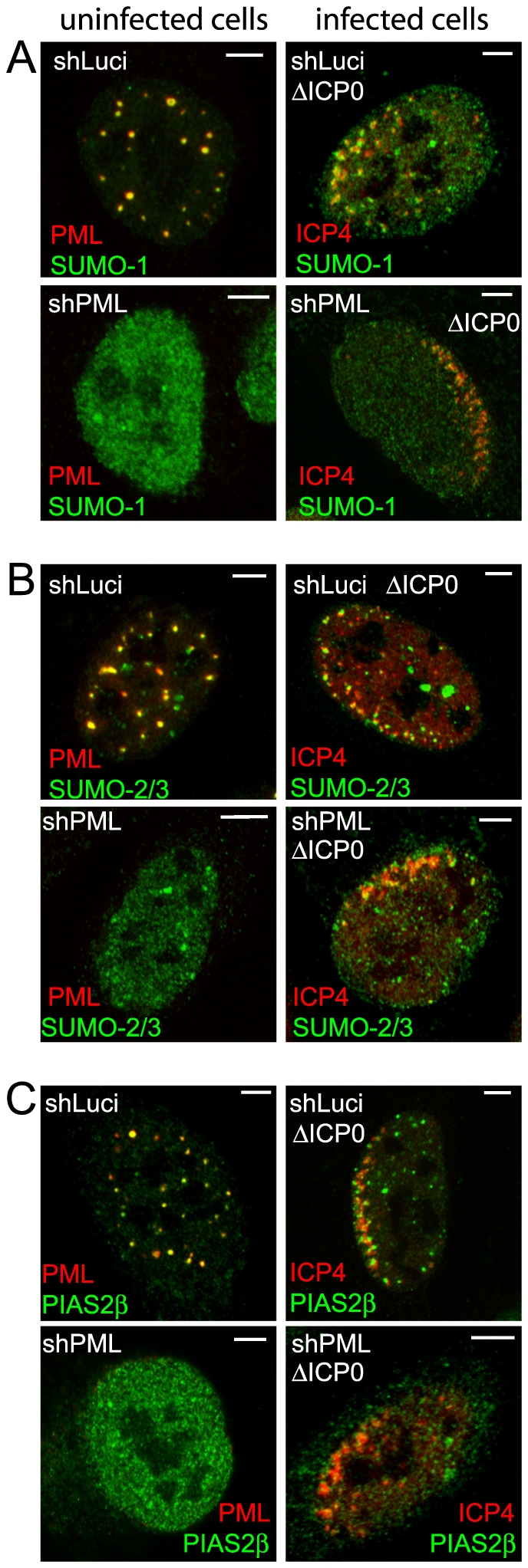
Recruitment of SUMO family members and PIAS2β to HSV-1 induced foci. Left-hand images show uninfected cells and the co-localization of SUMO-1 (A), SUMO-2/3 (B) and PIAS2β (C) (green) with PML (red) in control (upper rows of each block of 4 images) and PML depleted (low rows of each block of 4 images) HepaRG cells. Right-hand images show typical examples of recruitment of the indicated proteins to sites associated with HSV-1 genomes (ICP4; red) in cells at the edges of ICP0 null mutant (ΔICP0) plaques in control and PML depleted HepaRG cells. Scale bars indicate 5 µm.

The requirement of the SIMs of PML, Sp100 and hDaxx for recruitment to the HSV-1 induced foci suggests that these proteins may be interacting with components of the SUMO conjugation pathway at these locations. Therefore we examined the behaviour of SUMO isoforms and the SUMO E3 ligase PIAS2β in this experimental system. We found that PIAS2β, as detected by an antibody that recognizes the endogenous protein, is an ND10 component (Figure 9C, upper left). This suggests that PIAS proteins could be involved in ND10 assembly, consistent with the observation that ectopically expressed tagged PIAS1 localizes to ND10 in Vero cells [36]. In ICP0-null mutant HSV-1 infected cells, SUMO-1, SUMO-2/3 and PIAS2β were clearly recruited to the virus-induced foci (Figure 9A–C, right-hand panels). It could be argued that the presence of SUMO isoforms would be expected because both PML and Sp100 are recruited to the foci, and both are heavily SUMO modified. However, recruitment of SUMO-2/3 was readily evident in PML depleted cells (in which SUMO modification of Sp100 is highly compromised) (Figure 9B), and although recruitment of SUMO-1 and PIAS2β was very weak at best in PML-depleted HepaRG cells (Figures 9A, 9C and S11D), recruitment of PIAS2β remained strong in PML-depleted HFs (Figure S11C). The presence of a SUMO E3 ligase in the virus-induced foci suggests that the recruited SUMO-2/3 signal could include newly generated SUMO conjugates, and it is possible that this activity drives the PML-independent but SIM-dependent recruitment of proteins such as hDaxx. Consistent with this hypothesis, we have found that the recruitment of PML and SUMO isoforms requires Ubc9 (the sole SUMO E2 conjugating enzyme), and that the overall level of high molecular weight SUMO conjugates increases in ICP0 null mutant HSV-1 infections (C. Boutell, D. Cuchet-Lourenço, E. Vanni, A. Orr, and R.D. Everett, unpublished data).

### Recruitment of hDaxx and PML.I to HSV-1 induced foci correlates with their antiviral properties

**Figure 10:**
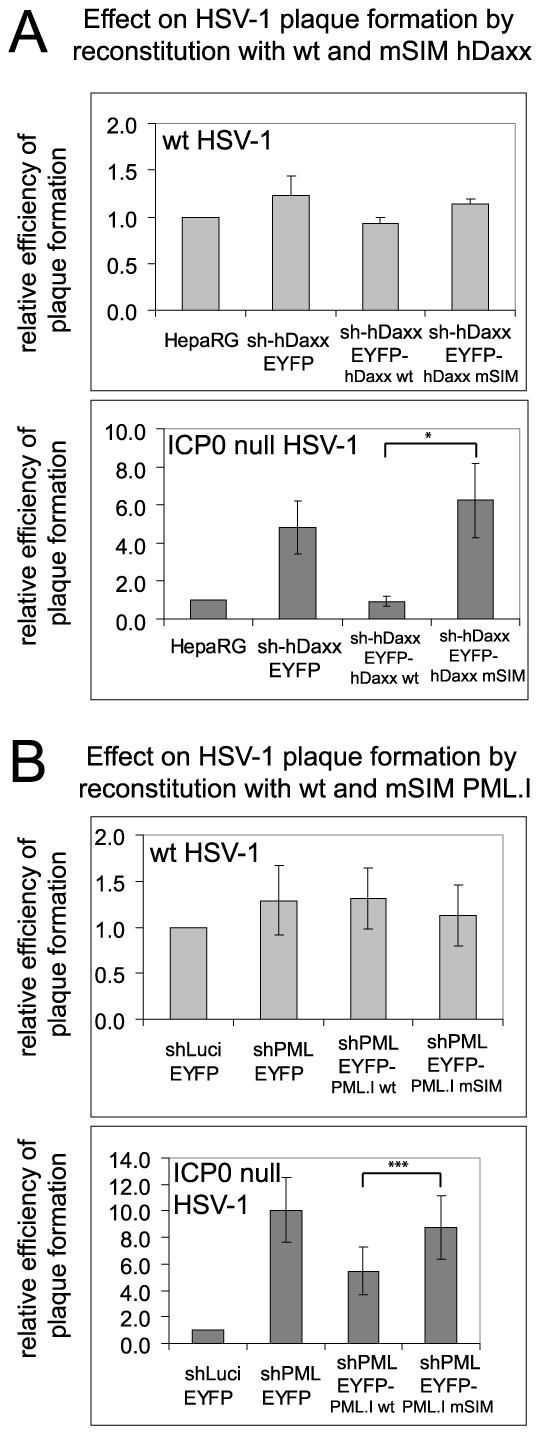
The SIMs of PML and hDaxx are required for repression of ICP0 null mutant HSV-1 plaque formation. A. Relative plaque forming efficiencies of wt (upper histogram) and ICP0-null mutant (lower histogram) HSV-1 in control and hDaxx-depleted cells and in cells reconstituted with the wt and mSIM mutant proteins. The data show means and standard deviations from 3 independent experiments. The statistical significance between the ICP0 null mutant plaque forming efficiencies in wt and mSIM mutant reconstituted cells is marked (Student's two-tailed t-Test, p = <0.05). B. As in A, except using control, PML-depleted and the indicated PML reconstituted cell lines. The data show means and standard deviations from 5 independent experiments. Statistical significance shown as in A (Student's two-tailed t-Test, p = <0.001).

We investigated the biological significance of the recruitment of PML and hDaxx to sites associated with HSV-1 genomes by comparing the plaque formation efficiencies of wt and ICP0 null mutant HSV-1 in various control and reconstituted cells. Depletion of either PML or hDaxx increases the plaque formation efficiency of ICP0 mutant HSV-1 while not affecting that of the wt [5], [6], [7]. As expected, the plaque forming efficiency of the wt virus was not significantly different in any of the cell lines tested here (Figures 10A and 10B, upper histograms). Increased plaque formation of ICP0 null mutant HSV-1 was observed in cells depleted of either PML or hDaxx, and reintroduction of PML.I and hDaxx reversed these phenotypes partially and completely, respectively (Figures 10A and 10B, lower histograms), confirming previous work [7], [23]. However, reintroduction of the mSIM mutants of PML.I and hDaxx had no effect on ICP0 null mutant HSV-1 plaque formation (Figures 10A and 10B, lower histograms). It may be relevant that mutation of its SIM reduces the transcriptional repression activity of hDaxx [35]. We have reported previously that PML.I.KK, PML.I.Δ7a are unable to reproduce the repressive effect of PML.I on ICP0 null mutant HSV-1 plaque formation [23]. The analogous experiments with Sp100 were not informative because reintroduction of Sp100A into Sp100 depleted cells did not reverse the effect of Sp100 depletion (data not shown), possibly because it is the longer isoforms of Sp100 (Sp100B, -C and -HMG) that are thought to act as repressors, rather than Sp100A [37], [38], [39]. There is therefore a correlation between the recruitment of PML and hDaxx to foci associated with HSV-1 genomes and their involvement in intrinsic resistance to virus infection.

## Discussion

The recruitment of ND10 components to sites that are closely associated with parental HSV-1 genomes and early replication compartments is a dramatic cellular response to entry of the viral genome into the nucleus. It occurs very rapidly, being detectable as early as 30 minutes after addition of virus to a cell monolayer [20], and it occurs independently of *de novo* viral protein synthesis, implying that it is the viral DNA itself (perhaps in association with viral tegument proteins) that signals the response [18]. These observations raise several important questions, including the nature of the mechanism underlying the recruitment, the biological significance of the response, and the wider implications of these events.

We found that endogenous PML and Sp100 have dominant influences on the behaviour of introduced mutant forms of these proteins, obscuring the role of the mutated motifs (Figures 1–3, 5 and 7). Expression of the reintroduced proteins at close to endogenous levels is also important, as when expressed in excess a protein may be unable to interact efficiently with limiting binding partners in the cell. With these issues overcome, we found that the recruitment of PML to the virus-induced foci depends on its SIM, despite the fact that the mSIM mutant is indistinguishable from the wt in terms of localization and SUMO modification in uninfected cells (Figure S6). Recruitment of PML to the virus-induced sites is also compromised by mutation of the major SUMO modification sites (K160 and K490) and by alterations in the B-Box 1, coiled-coil and RING motifs (Figures 3 and 5). It is unsurprising that elements of the TRIM are important for PML behaviour because these mutations greatly reduce SUMO modification [23] and are likely to have major consequences on PML structure and interactions.

It has been reported that the SIM of PML is required for normal ND10 assembly [26]. Our finding that PML.I.mSIM and PML.I.Δ7a co-localize with Sp100 in PML depleted cells is not inconsistent with the data in this previous study, since the effect of removal of the SIM was observed only when the PML isoform used (PML.III) also lacked SUMO modification sites. Thus the SIM mutant of PML.III still co-localized with hDaxx and SUMO, even in PML (−/−) mouse fibroblasts [26]. That the SIM is not essential for ND10 localization is also consistent with previous studies utilizing PML.VI in PML depleted cells [8], [23], because this is a natural SIM deletion mutant.

The SIMs of both Sp100 and hDaxx are also important for their recruitment to the virus-induced foci (Figures 7 and 8). Given that SUMO modification of Sp100A is not required (Figures 6 and 7), and hDaxx appears not to be detectably SUMO modified in our system (Figure 8), we conclude that it is the SIM rather than SUMO modification that is the common essential feature for recruitment of these proteins to the virus-induced foci. The conclusion that SUMO modification of Sp100A is not required for this behaviour is supported by the observation that Sp100 is not SUMO modified in PML depleted cells, yet is still recruited to the viral foci [5], [6].

The importance of the SIM in recruitment to the virus-induced foci implies that PML, hDaxx and Sp100 are responding to an earlier SUMO-dependent event at these sites. This conclusion is supported by our recent data demonstrating that Ubc9 is required for efficient recruitment of PML to the virus induced foci (C. Boutell, D. Cuchet-Lourenco, E. Vanni, A. Orr, and R.D. Everett, unpublished data). This raises the question of which factors these ND10 components are interacting with, through their SIMs, to enable their recruitment to the virus-induced foci. Recruitment of PML is not dependent on *de novo* viral gene expression [18], implying that the initial event involves cellular proteins and their recognition of the viral genomes. Because the viral DNA is not chromatinized at the time of entry into the nucleus, its conformation is entirely different from cellular chromatin. It would be expected that many cellular proteins would become associated with the initially naked viral DNA, and one or more of these could initiate the SUMO-dependent recruitment process. The detection of PIAS2β in the novel foci implies that SUMO E3 ligases are involved in the assembly of the novel foci. This is the first report that PIAS2β is involved in ND10 biology, and it is intriguing that the protein has also been implicated in DNA damage and interferon pathways [40], [41].

The mechanisms that underlie formation of the virus-induced structures are distinct in a number of respects from those required for normal ND10 assembly. For example, PML is not required for recruitment of Sp100 and hDaxx to the virus-induced foci [5], [6], [20], and depletion of any one of these three proteins does not eliminate recruitment of the remaining two [6], [7]. Some of the PML mutants also illustrate the differences between normal and virus-induced ND10 related structures: PML.I.ΔBB2 does not colocalize with Sp100 in uninfected cells [23] but is recruited very efficiently to the virus-induced foci (Figure 5). On the other hand, recruitment of ATRX to both normal and virus-induced ND10 structures is dependent on hDaxx [7], illustrating that both specific protein-protein interactions and SIM dependent interactions are involved in building the virus-induced foci. PML-PML interactions through the coiled-coil and Sp100-Sp100 interactions through the HSR motif also influence assembly of the virus-induced structures (Figures 2, 3 and 7). A picture emerges that the building of these foci involves multiple interactions between distinct proteins, protein dimerization events, and SIM-SUMO interactions.

Our data imply a general importance for SUMO related pathways in the nucleation of the HSV-1 induced ND10-like foci. Because SUMO-2/3 and PIAS2β can also be recruited into these structures in a PML-independent manner (Figures 9 and S11), it is attractive to speculate that these events reflect ongoing SUMO conjugation events. We note that the genomes and replication compartments of many DNA viruses are closely associated with PML and ND10-like structures (reviewed in [42], [43]). It would be surprising if the principles revealed here concerning HSV-1 infection were not involved in ND10 association with other viral genomes, and there is evidence that this is the case in human cytomegalovirus infected cells [8], [44]. Therefore the events reported here are likely to reflect a more general cellular response to foreign DNA entering the nucleus. It is possible that the cell is responding to viral genomes in a manner related to that of the DNA damage response. Recent evidence demonstrates that SUMO modification is intimately involved in the assembly of DNA damage response foci, and SUMO conjugates are present at these locations [45], [46]. ICP0 inhibits the formation of these structures by inducing the degradation of RNF8 and RNF168 [47], but it is possible that ICP0 might also impede any recruitment of SUMO conjugated proteins to DNA damage foci, as it does in regard to the viral genome associated ND10-like foci (see below). These observations raise the intriguing possibility of commonalities between the DNA damage response, the assembly of HSV-1 induced ND10-like foci, and intrinsic resistance to HSV-1 infection.

The observations that the SIM mutants of PML.I and hDaxx are unable to reproduce the repression of ICP0 null mutant HSV-1 infection conferred by the wt proteins (Figure 10) imply that the recruitment of these proteins to the virus-induced foci is biologically significant and contributes to the repression of ICP0 null mutant HSV-1 infection. Recruitment of all ND10 component proteins so far studied is counteracted by ICP0 [7], [18], [20]. ICP0 inhibits PML recruitment by inducing its degradation [15], [17]. This simple mechanism does not, however, explain why recruitment of all ND10 proteins is inhibited by ICP0. Although ICP0 promotes the loss of SUMO modified Sp100 [16] this cannot explain why Sp100 recruitment is inhibited because the K297R mutant is still recruited (Figure 7). Furthermore, ICP0 does not promote the degradation of either hDaxx or ATRX [7]. Previous work has demonstrated that ICP0 induces the widespread degradation of SUMO conjugated proteins [15]. This activity provides an attractive explanation of how ICP0 inhibits recruitment of this group of proteins to virus-induced foci, since degradation of SUMO conjugates would eliminate SIM dependent interactions.

These arguments suggest a direct link between SUMO-dependent pathways and the mechanism of intrinsic cellular resistance to HSV-1 infection that is counteracted by ICP0. We propose that the cell responds to foreign DNA that enters the nucleus by stimulating SUMO conjugation events at sites associated with the introduced DNA, leading to recruitment of other proteins in a SIM dependent manner and resulting in a repressive environment. We note that there are several examples of factors involved in transcriptional repression that are regulated by SUMO modification [48], and that SUMO modification pathways have been linked to a general cellular response to pathogens [49]. This concept is strengthened by our related studies (C. Boutell, D. Cuchet-Lourenço, E. Vanni, A. Orr, and R.D. Everett, unpublished data) that demonstrate the involvement of Ubc9 in intrinsic antiviral resistance, and that ICP0 has SUMO-targeted ubiquitin ligase activities that play an important role in its ability to counteract this resistance.

## Materials and Methods

### Cells

U2OS, HEK-293T and human fibroblast cells were grown in Dulbecco's Modified Eagles' Medium with 10% fetal calf serum (FCS). Baby hamster kidney (BHK) cells were grown in Glasgow Modified Eagles' Medium with 10% new born calf serum and 10% tryptose phosphate broth. HepaRG cells [50] were grown in William's Medium E with 10% fetal bovine serum Gold (PAA Laboratories Ltd), 2 mM glutamine, 5 µg/ml insulin and 0.5 µM hydrocortisone. All cell growth media contained 100 units/ml penicillin and 100 µg/ml streptomycin.

### Lentivirus expression vectors and cells

Lentivirus vector plasmids expressing shRNAs, EYFP-hDaxx, and EYFP-PML isoforms I to VI, mutants of PML.I with lesions in the RING finger (ΔRING), B-Box 1 (ΔBB1), B-Box 2 (ΔBB2), the coiled-coil motif (ΔCC) and at SUMO modification sites K160 and K490 were as described [6], [7], [23]. The following PML mutants were constructed using PCR splicing with mutagenic oligonucleotides: PML.I.Δ7a and PML.IV.Δ7a (precise deletions of exon 7a in PML.I and PML.IV cDNAs); PML.IV.KK (K160R, K490R mutations in the PML.IV background); PML.I.mSIM (residues 566–569 VVVI mutated to VGGG); PML.I.K123 (mutations K65R, K160R, K490R); PML.I.K234 (K160R, K490R, K616R); PML.I.K1-4 (lysine residues K65, K160, K490 and K160 mutated to arginine). Lentivirus transduced HepaRG cells expressing EYFP-linked proteins were sorted by FACS, as described [23]. HFs expressing control and anti-PML shRNAs [5] and EYFP-PML.I, PML.VI, PML.I.Δ7a, PML.I.KK and PML.I.K1-4 were isolated using the same methodology.

### Sp100 and hDaxx expression vectors and cells

Lentivirus vectors expressing EYFP-Sp100 isoform A (using a cDNA resistant to the anti-Sp100 shRNA) and derivatives lacking the HSR region (Sp100.ΔHSR, deletion of residues 68–146), the major SUMO modification site and the SIM combined (Sp100.ΔSSIM, deletion of residues 289–327), the major SUMO modification site alone (Sp100.K297R) and with point mutations in the SIM alone (residues 323 to 326 IIVI changed to IGAG; Sp100.mSIM) were constructed using PCR splicing. A lentivirus vector expressing EYFP- hDaxx with lesions in the SIM (residues 733 to 736 IIVI changed to IGAG) was constructed by PCR directed mutagenesis. HepaRG cells expressing wt and mutant hDaxx were enriched by FACS.

### Lentivirus transduction

Lentivirus vector plasmids were co-transfected into HEK-293T cells with helper plasmids pVSV-G and pCMV.DR.8.91, then the supernatants were used to transduce HF or HepaRG cells. All shRNA vectors express puromycin for selection (initially 1 µg/ml, then reduced to 0.5 µg/ml during subsequent passage), and all expressing a protein of interest confer G418 resistance (selection initially 1 mg/ml, then reduced to 0.5 mg/ml during subsequent passage).

### HSV-1 strains and plaque assays

Wild type HSV-1 strain 17syn+ and its ICP0 null mutant derivative *dl*1403 [51] were grown in BHK cells and titrated in U2OS cells. Derivatives of wt and ICP0-null mutant HSV-1 that include a β-galactosidase gene linked to the human cytomegalovirus immediate-early promoter/enhancer (*in*1863 and *dl*1403/CMV*lac*Z) were used for plaque assays, as described [5].

### Western blot analysis and antibodies

Cells in 24-well dishes at 1×10^5^ cells per well were washed with phosphate buffered saline PBS) before harvesting in SDS-PAGE loading buffer. Proteins were resolved on 7.5% SDS-gels, then transferred to nitrocellulose membranes by western blotting. The following antibodies were used: anti-actin mAb AC-40 (Sigma-Aldrich); anti-PML mAb 5E10 [52]; anti-Sp100 rabbit serum SpGH [53]; anti-hDaxx rabbit polyclonal D7801 (Sigma-Aldrich); anti-EGFP rabbit polyclonal ab290 (Abcam).

### Immunofluorescence and confocal microscopy

Cells on 13 mm glass coverslips were fixed using 1.5% (v/v) formaldehyde in PBS containing 2% sucrose then treated with 0.5% Nonidet P40 substitute (EuroClone S.p.A.) in PBS/10% sucrose. PML was detected with mAb 5E10 and ICP4 with mAb 58S. Rabbit polyclonal antibodies were used for Sp100 (SpGH), hDaxx (07-471, Upstate), SUMO-1 (ab32058, Abcam), SUMO-2/3 (ab3742-100, Abcam), PIAS2β (gifted by Mary Dasso). The secondary antibodies were FITC conjugated goat anti-mouse IgG (Sigma), Alexa 488 and Alexa 633 conjugated goat anti-rabbit and anti-mouse IgG, and Alexa 555 conjugated donkey anti-rabbit and anti-mouse IgG, (Invitrogen). A glycerol-based mounting medium was used (Citifluor AF1). The samples were examined using a Zeiss LSM 510 confocal microscope with 488 nm, 543 nm and 633 nm laser lines and a ×63 Plan-Apochromat oil immersion lens, NA 1.40. Exported images were processed using Adobe Photoshop with minimal adjustment, then assembled for presentation using Adobe Illustrator.

### Fluorescence recovery after photobleaching

Cells were seeded into modified 35 mm dishes with the central area replaced by coverslip glass (MatTek Corporation). Fluorescence recovery after photobleaching (FRAP) was conducted using an LSM 510 META microscope with full environmental control. In each experiment, 3 PML foci in 10 different cells were subject to bleaching (100% power of the 514 nm laser, 10 reiterations), then 20 images were captured over a period of approximately two minutes during the recovery phase. Regions of interest were analyzed by subtracting the average pixel intensity in unbleached background areas and normalizing to any changes in overall intensity of a similar unbleached PML structure to control for any bleaching of the scanned areas during image acquisition. The data for each protein were assembled into an Excel file for graphical representation of the average plus standard deviation at each time point.

## Supporting Information

Figure S1 Recruitment of PML isoforms I to VI in control shLuci HepaRG cells. The separated channels (shown in greyscale) and the merge of the images in Figure 1C are shown. The background cell type, the identity of the EYFP-linked PML isoform proteins and the colours used for the merged channels are indicated on each set of panels. For comparison, the upper left two rows of the upper left block of images show typical examples in which recruitment does occur. Scale bars indicate 5 µm.
(PDF)

Figure S2 Recruitment of PML isoforms I to VI in PML depleted HepaRG cells. The separated channels (shown in greyscale) and the merge of the images in Figure 1D are shown. The background cell type, the identity of the EYFP-linked PML isoform proteins and the colours used for the merged channels are indicated on each set of panels. For comparison, the upper left two rows of the upper left block of images show typical examples in which recruitment does occur. Scale bars indicate 5 µm.
(PDF)

Figure S3 Confocal microscopy analysis of control and PML depleted HFs expressing EYFP-PML.I and EYFP-PML.I.Δ7a. A. Control shLuci expressing transduced HFs and derivatives expressing EYFP-PML.I and the PML.I.Δ7a mutant. The upper row shows HF-shLuci control cells stained for endogenous PML and Sp100. The introduced PML.I and PML.I.Δ7a proteins were detected by EYFP autofluorescence and staining for Sp100 (red). B. As A, but in the PML-depleted HF-shPML background. The background cell type, the identity of the detected proteins and the colours used for the merged channels are indicated on each set of panels. Scale bars indicate 5 µm.
(PDF)

Figure S4 Comparative data on recruitment of PML.I and PML.VI in HFs. A. HF-shLuci cells infected with ICP0 null mutant HSV-1 (ΔICP0). The images are of cells at the periphery of developing plaques, showing assays of recruitment PML proteins to sites associated with viral genomes (ICP4, red). Upper row; endogenous PML; middle row, introduced EYFP-PML.I; lower row, introduced EYFP-PML.VI. B. As in A, but in the PML-depleted HF-shPML background. The background cell type, the identity of the detected proteins and the colours used for the merged channels are indicated on each set of panels. Scale bars indicate 5 µm.
(PDF)

Figure S5 PML.I.Δ7a is not recruited to virus-induced foci in the absence of endogenous PML. A. The separated greyscale and merged channels from Figure 2D. B and C. Comparative data in the HF background. Recruitment of EYFP-PML.I.Δ7a to virus-induced sites in the HF-shLuci (B) but not the HF-shPML (C) reconstituted cells. The background cell type, the identity of the detected proteins and the colours used for the merged channels are indicated on each set of panels. Scale bars indicate 5 µm.
(PDF)

Figure S6 Analysis of mutant protein PML.I.mSIM. A. Map of PML.I showing the location and nature of the mSIM mutation. B. Western blot analysis of EYFP-PML.I and EYFP-PML.I.mSIM in control and PML depleted backgrounds, detected with an anti-EGFP antibody. The locations of the unmodified PML bands are indicated by asterisks. C. Immunofluorescence analysis of EYFP-PML.I.mSIM in uninfected control and PML depleted cells stained for Sp100 (red). The images are single plane projections from short z-stacks. D. Immunofluorescence analysis of EYFP-PML.I.mSIM in developing ICP0-null mutant HSV-1 plaques in control and PML depleted cells. Scale bars indicate 5 µm.
(PDF)

Figure S7 Analysis of SUMO modification site mutants of PML.I. A. Map of PML.I showing the four lysine residues of interest and nomenclature of the mutant proteins. B. Western blot analysis of PML.I SUMO modification site mutants in control and PML depleted cells.
(PDF)

Figure S8 Confocal microscopy analysis of SUMO modification mutants of PML.I and PML.IV. A. Immunofluorescence analysis of EYFP-PML.I.4KR in uninfected control and PML depleted HepaRG cells. B. The separated greyscale and merged channels from the images of Figure 3D. C. EYFP-PML.I.4KR is not recruited to viral foci in ICP0 null mutant infected PML depleted cells. The images are single plane projections from short z-stacks. Scale bars indicate 5 µm.
(PDF)

Figure S9 Comparative confocal microscopy analysis of SUMO modification mutants of PML.I in HFs. A. Immunofluorescence analysis of EYFP-PML.I.4KR in uninfected HF-shLuci cells and of EYFP-PML.I.KK and EYFP-PML.I.4KR in uninfected HF-shPML cells stained for Sp100. B. Immunofluorescence analysis of EYFP-PML.I.4KR in ICP0-null mutant HSV-1 infected HF-shLuci cells and of EYFP-PML.I.KK and EYFP-PML.I.4KR in ICP0-null mutant HSV-1 infected HF-shPML cells. The images are single plane projections from short z-stacks. Scale bars indicate 5 µm.
(PDF)

Figure S10 Separated channels of images depicting failure of TRIM mutants of PML.I (A), Sp100 (B) and hDaxx (C) to be recruited to sites associated with HSV-1 genomes and early replication compartments. The two colour images are taken from the indicted figures in the main text, with the greyscale images of their separated channels. The background cell type, the identity of the detected proteins and the colours used for the merged channels are indicated on each set of panels. Scale bars indicate 5 µm.
(PDF)

Figure S11 Recruitment of SUMO family members and PIAS2β to HSV-1 induced foci in control and PML-depleted HFs. Left-hand images of each block of 4 in parts A–C show uninfected cells and the co-localization of SUMO-1 (A), SUMO-2/3 (B) and PIAS2β (C) (green) with PML (red) in control (upper rows of each block of 4 images) and PML depleted (lower rows of each block of 4 images) HFs. Right-hand images show typical examples of recruitment of the indicated proteins to sites associated with HSV-1 genomes (ICP4; red) in cells at the edges of ICP0 null mutant (ΔICP0) plaques in control and PML depleted HFs. Panel D shows the separated channels of the images of Figure 9 panels A and C, illustrating SUMO-1 and PIASβ in infected PML depleted HepaRG cells. Scale bars indicate 5 µm.
(PDF)
