# Supplementary figures and images for: SUMO Pathway Dependent Recruitment of Cellular Repressors to Herpes Simplex Virus Type 1 Genomes
Source: PLoS Pathog. 2011 Jul 14;7(7):e1002123. doi: 10.1371/journal.ppat.1002123 (PMC3136452; doi:10.1371/journal.ppat.1002123)

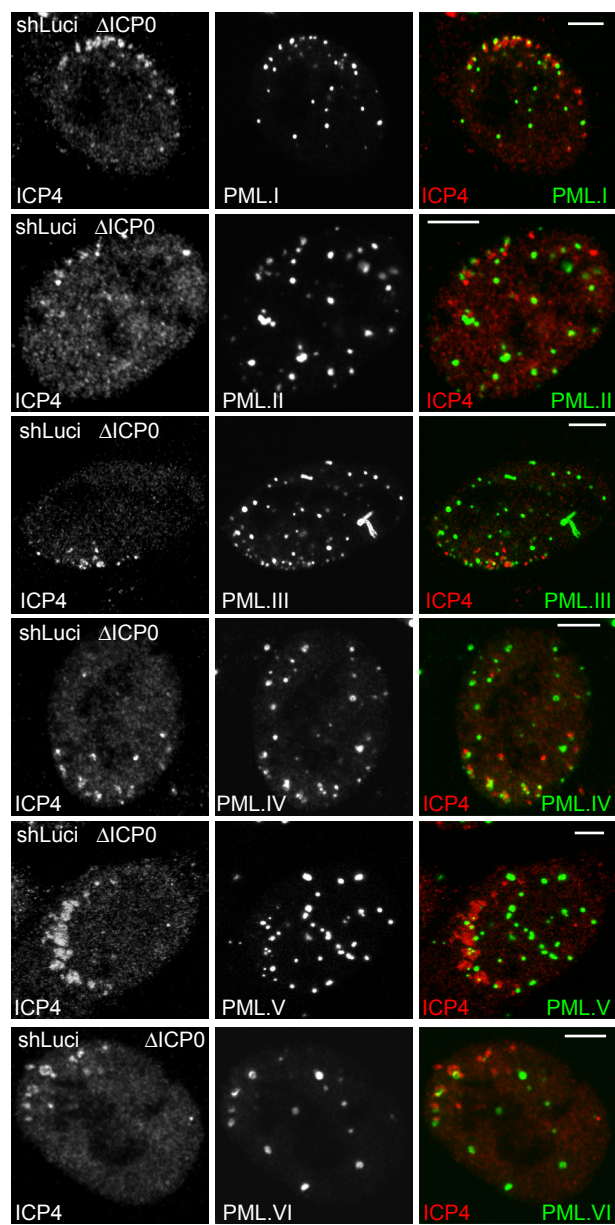

Supplement: Figure S1 — Recruitment of PML isoforms I to VI in control shLuci HepaRG cells. The separated channels (shown in greyscale) and the merge of the images in Figure 1C are shown. The background cell type, the identity of the EYFP-linked PML isoform proteins and the colours used for the merged channels are indicated on each set of panels. For comparison, the upper left two rows of the upper left block of images show typical examples in which recruitment does occur. Scale bars indicate 5 µm. (PDF) [file ppat.1002123.s001.pdf]

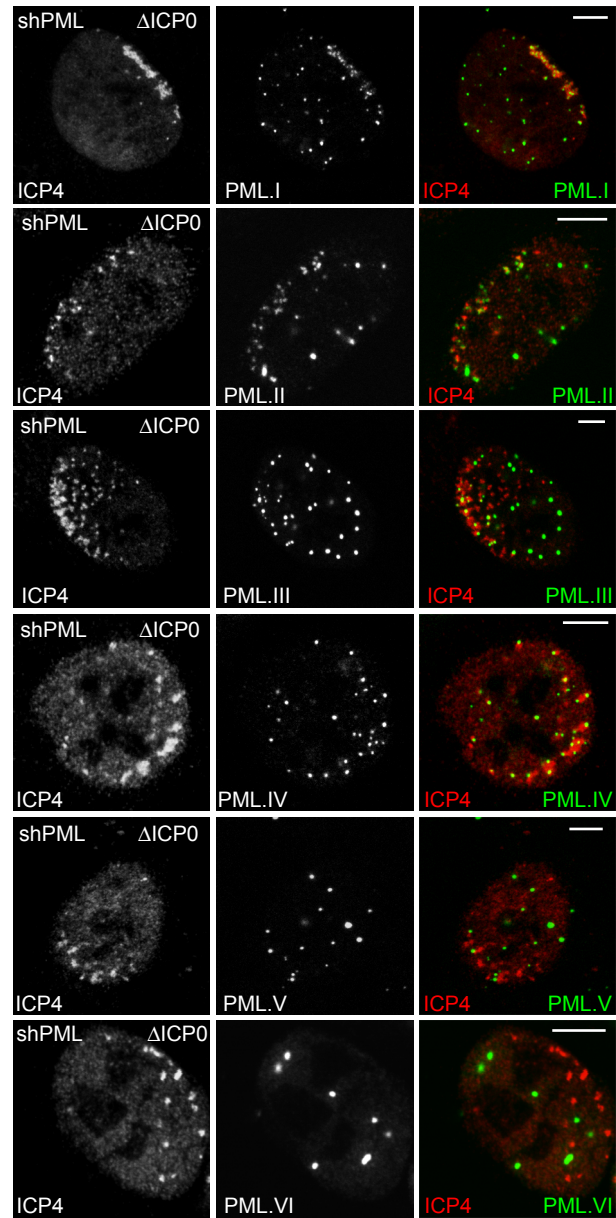

Supplement: Figure S2 — Recruitment of PML isoforms I to VI in PML depleted HepaRG cells. The separated channels (shown in greyscale) and the merge of the images in Figure 1D are shown. The background cell type, the identity of the EYFP-linked PML isoform proteins and the colours used for the merged channels are indicated on each set of panels. For comparison, the upper left two rows of the upper left block of images show typical examples in which recruitment does occur. Scale bars indicate 5 µm. (PDF) [file ppat.1002123.s002.pdf]

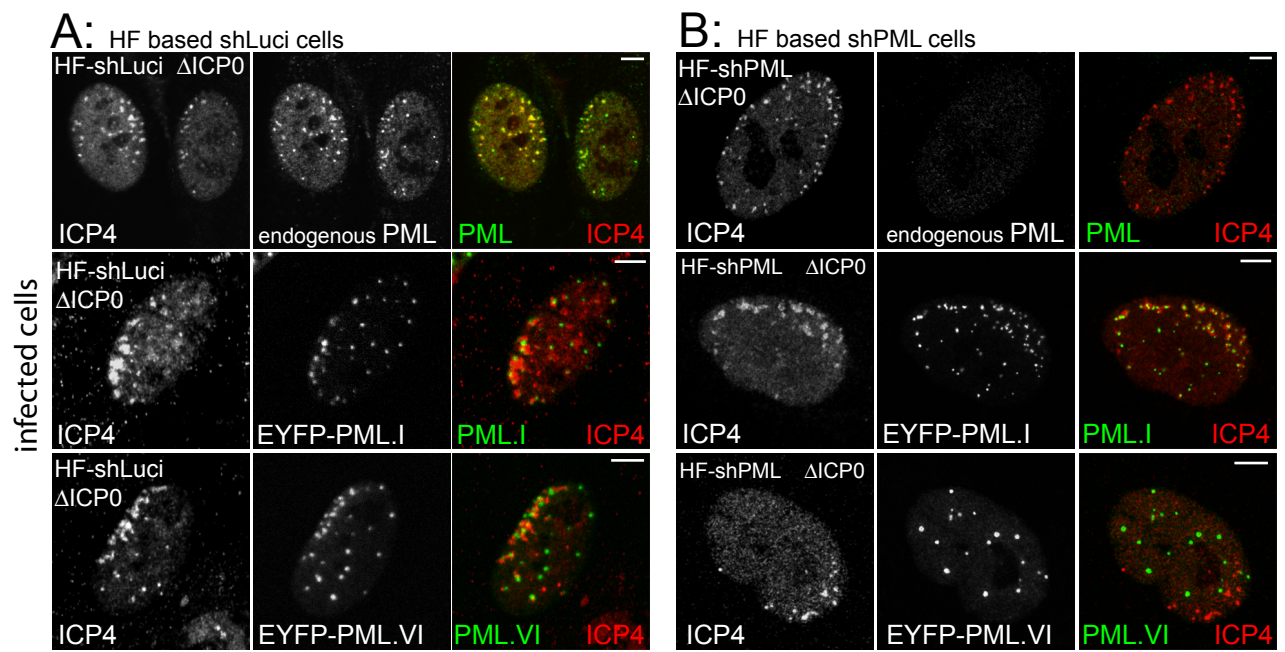

Supplement: Figure S4 — Comparative data on recruitment of PML.I and PML.VI in HFs. A. HF-shLuci cells infected with ICP0 null mutant HSV-1 (ΔICP0). The images are of cells at the periphery of developing plaques, showing assays of recruitment PML proteins to sites associated with viral genomes (ICP4, red). Upper row; endogenous PML; middle row, introduced EYFP-PML.I; lower row, introduced EYFP-PML.VI. B. As in A, but in the PML-depleted HF-shPML background. The background cell type, the identity of the detected proteins and the colours used for the merged channels are indicated on each set of panels. Scale bars indicate 5 µm. (PDF) [file ppat.1002123.s004.pdf]

A: From Figure 2D

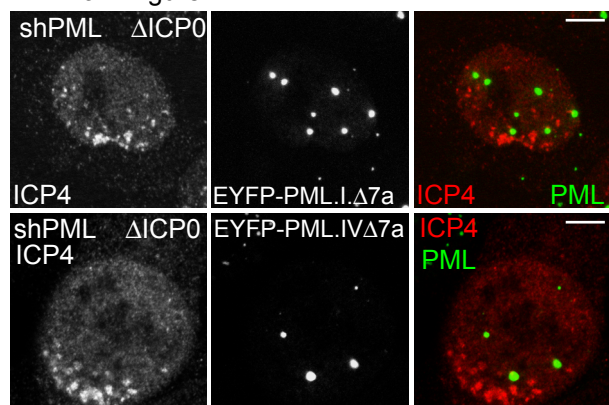

B: HF based shLuci cells

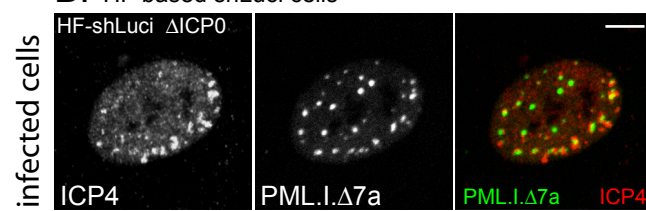

C: HF based shPML cells

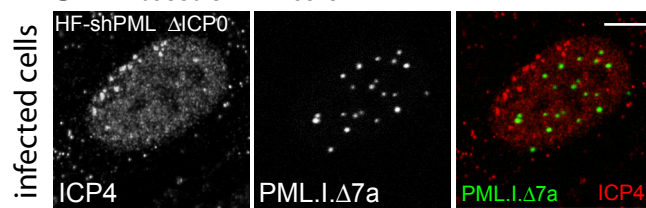

Supplement: Figure S5 — PML.I.Δ7a is not recruited to virus-induced foci in the absence of endogenous PML. A. The separated greyscale and merged channels from Figure 2D. B and C. Comparative data in the HF background. Recruitment of EYFP-PML.I.Δ7a to virus-induced sites in the HF-shLuci (B) but not the HF-shPML (C) reconstituted cells. The background cell type, the identity of the detected proteins and the colours used for the merged channels are indicated on each set of panels. Scale bars indicate 5 µm. (PDF) [file ppat.1002123.s005.pdf]

**A**

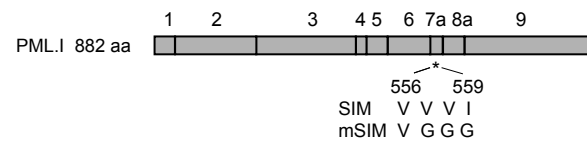

**B**

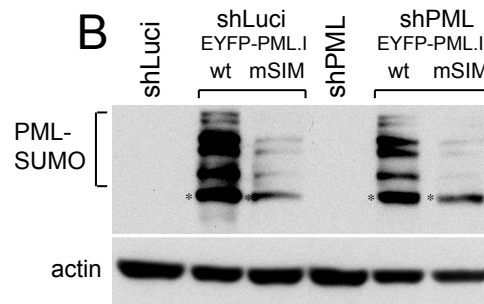

**C:** uninfected HepaRG based cells

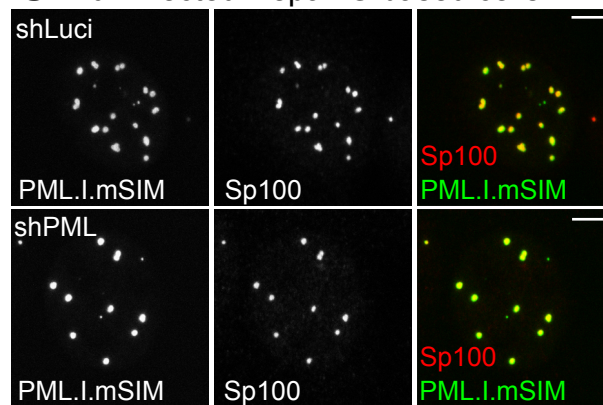

**D:** infected HepaRG based cells

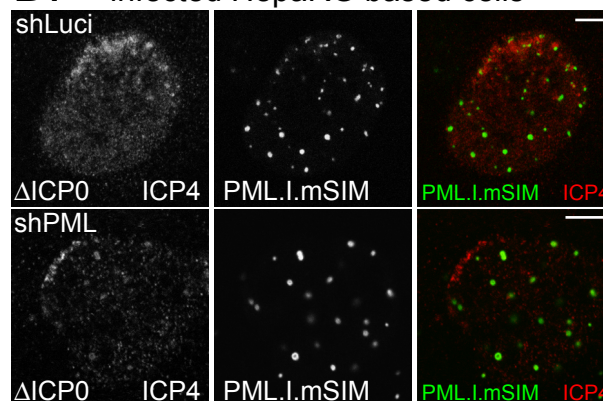

Supplement: Figure S6 — Analysis of mutant protein PML.I.mSIM. A. Map of PML.I showing the location and nature of the mSIM mutation. B. Western blot analysis of EYFP-PML.I and EYFP-PML.I.mSIM in control and PML depleted backgrounds, detected with an anti-EGFP antibody. The locations of the unmodified PML bands are indicated by asterisks. C. Immunofluorescence analysis of EYFP-PML.I.mSIM in uninfected control and PML depleted cells stained for Sp100 (red). The images are single plane projections from short z-stacks. D. Immunofluorescence analysis of EYFP-PML.I.mSIM in developing ICP0-null mutant HSV-1 plaques in control and PML depleted cells. Scale bars indicate 5 µm. (PDF) [file ppat.1002123.s006.pdf]

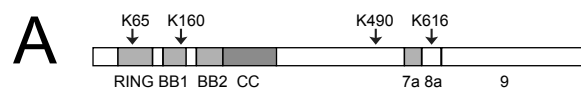

KK: K160R, K490R  
 K234: K160R, K490R, K616R  
 K123: K65R, K160R, K490R  
 4KR: K65R, K160R, K490R, K616R

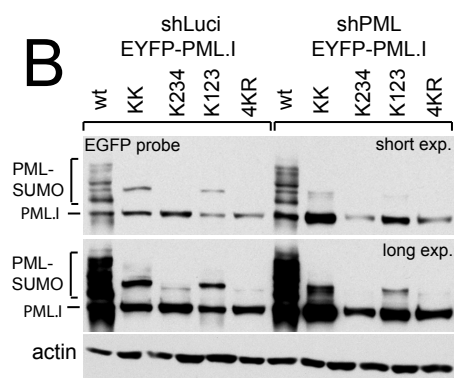

Supplement: Figure S7 — Analysis of SUMO modification site mutants of PML.I. A. Map of PML.I showing the four lysine residues of interest and nomenclature of the mutant proteins. B. Western blot analysis of PML.I SUMO modification site mutants in control and PML depleted cells. (PDF) [file ppat.1002123.s007.pdf]

**A:** uninfected HepaRG based cells

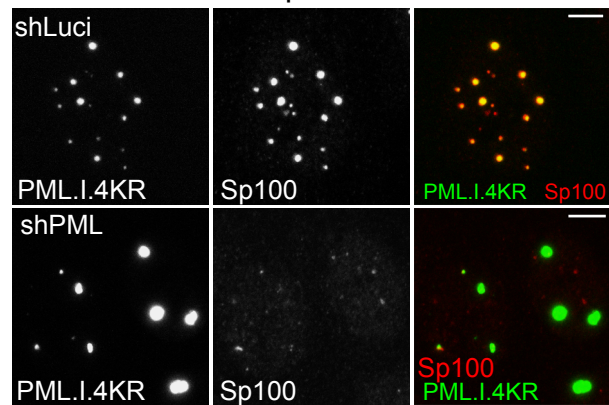

**B:** From Figure 3D

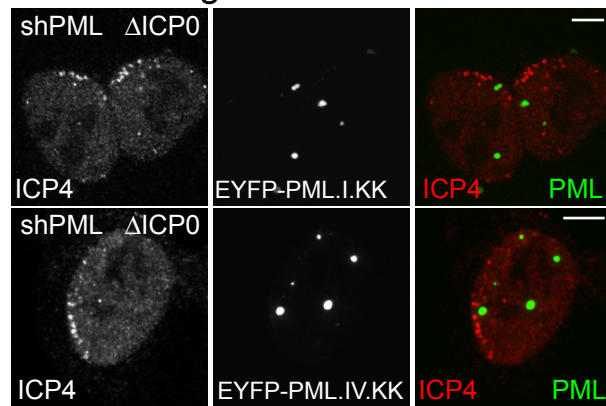

**C:** infected HepaRG based cells

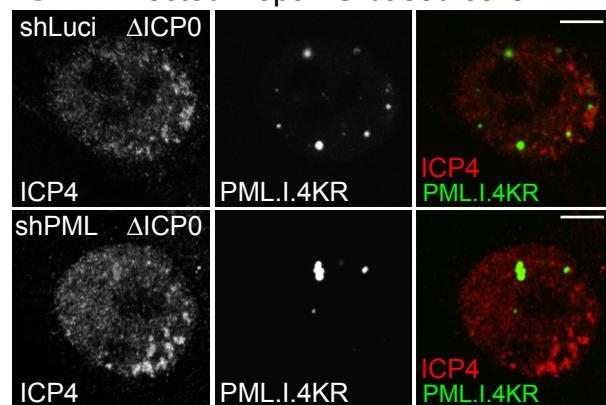

Supplement: Figure S8 — Confocal microscopy analysis of SUMO modification mutants of PML.I and PML.IV. A. Immunofluorescence analysis of EYFP-PML.I.4KR in uninfected control and PML depleted HepaRG cells. B. The separated greyscale and merged channels from the images of Figure 3D. C. EYFP-PML.I.4KR is not recruited to viral foci in ICP0 null mutant infected PML depleted cells. The images are single plane projections from short z-stacks. Scale bars indicate 5 µm. (PDF) [file ppat.1002123.s008.pdf]

**A:** uninfected HF based cells

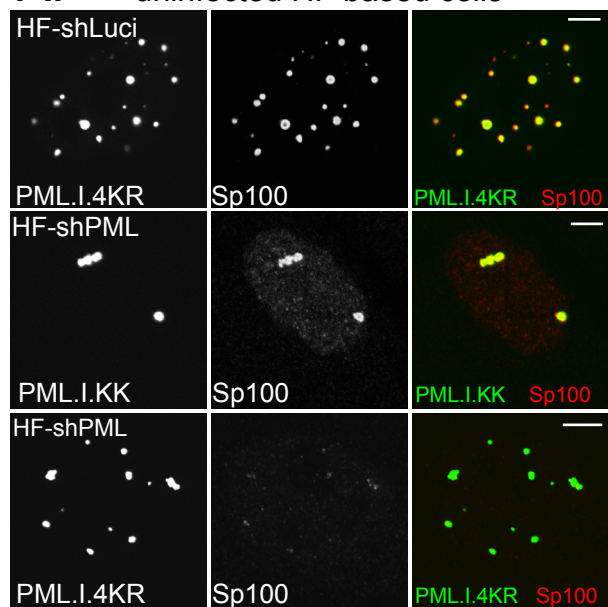

**B:** infected HF based cells

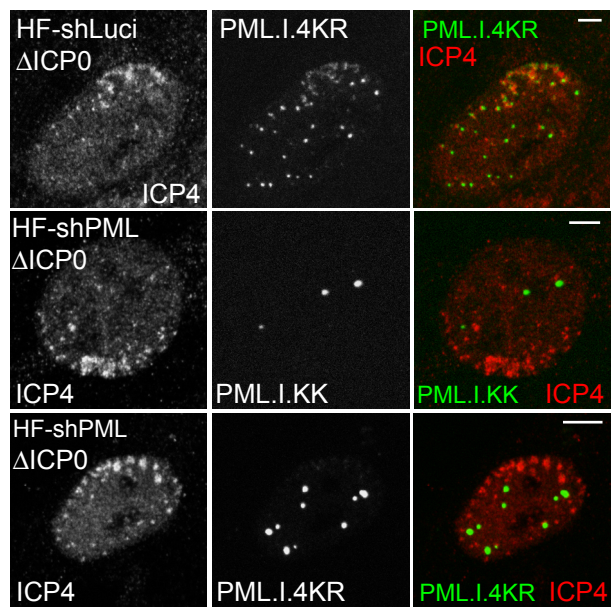

Supplement: Figure S9 — Comparative confocal microscopy analysis of SUMO modification mutants of PML.I in HFs. A. Immunofluorescence analysis of EYFP-PML.I.4KR in uninfected HF-shLuci cells and of EYFP-PML.I.KK and EYFP-PML.I.4KR in uninfected HF-shPML cells stained for Sp100. B. Immunofluorescence analysis of EYFP-PML.I.4KR in ICP0-null mutant HSV-1 infected HF-shLuci cells and of EYFP-PML.I.KK and EYFP-PML.I.4KR in ICP0-null mutant HSV-1 infected HF-shPML cells. The images are single plane projections from short z-stacks. Scale bars indicate 5 µm. (PDF) [file ppat.1002123.s009.pdf]

A: From Figure 5B

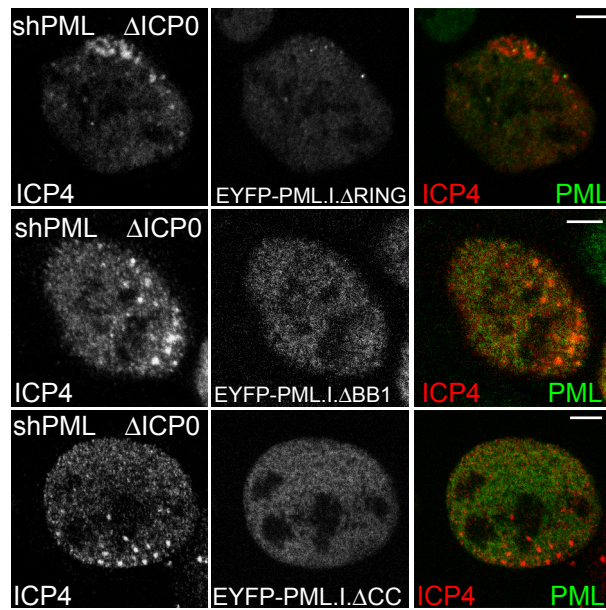

B: From Figure 7

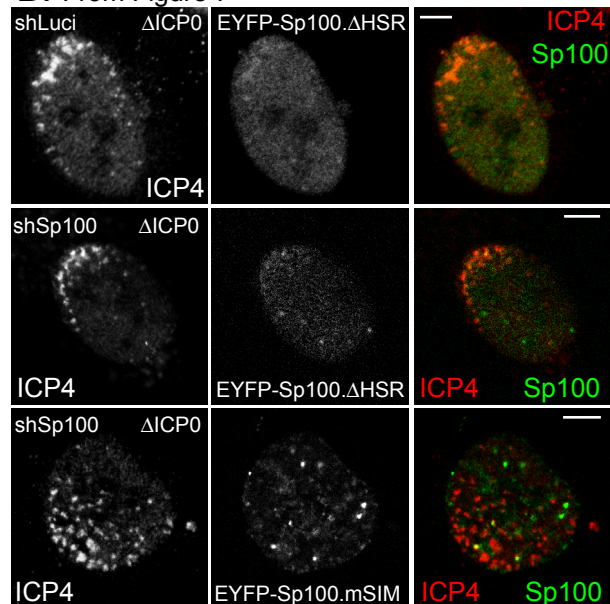

C: From Figure 8

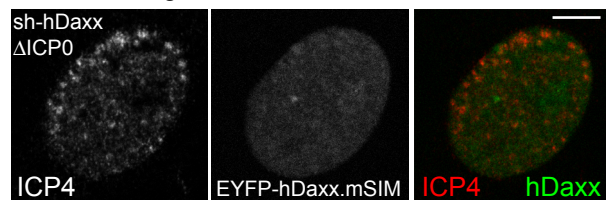

Supplement: Figure S10 — Separated channels of images depicting failure of TRIM mutants of PML.I (A), Sp100 (B) and hDaxx (C) to be recruited to sites associated with HSV-1 genomes and early replication compartments. The two colour images are taken from the indicted figures in the main text, with the greyscale images of their separated channels. The background cell type, the identity of the detected proteins and the colours used for the merged channels are indicated on each set of panels. Scale bars indicate 5 µm. (PDF) [file ppat.1002123.s010.pdf]

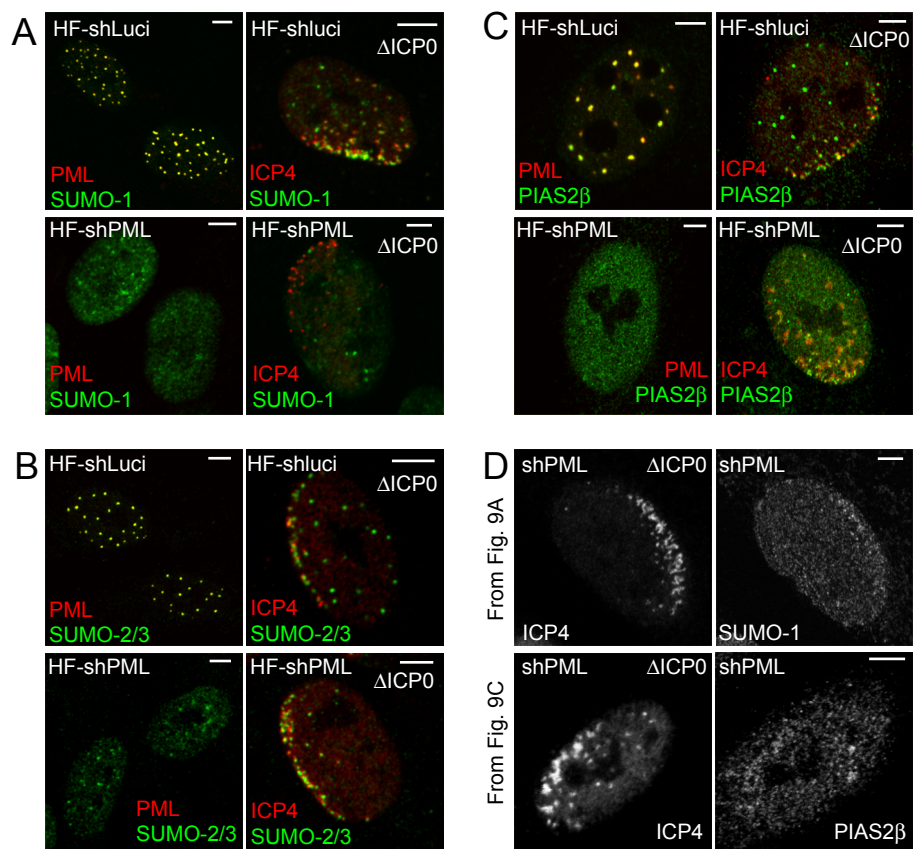

Supplement: Figure S11 — Recruitment of SUMO family members and PIAS2β to HSV-1 induced foci in control and PML-depleted HFs. Left-hand images of each block of 4 in parts A–C show uninfected cells and the co-localization of SUMO-1 (A), SUMO-2/3 (B) and PIAS2β (C) (green) with PML (red) in control (upper rows of each block of 4 images) and PML depleted (lower rows of each block of 4 images) HFs. Right-hand images show typical examples of recruitment of the indicated proteins to sites associated with HSV-1 genomes (ICP4; red) in cells at the edges of ICP0 null mutant (ΔICP0) plaques in control and PML depleted HFs. Panel D shows the separated channels of the images of Figure 9 panels A and C, illustrating SUMO-1 and PIASβ in infected PML depleted HepaRG cells. Scale bars indicate 5 µm. (PDF) [file ppat.1002123.s011.pdf]
