# Supplementary material for: SUMO Pathway Dependent Recruitment of Cellular Repressors to Herpes Simplex Virus Type 1 Genomes
Source: PLoS Pathog. 2011 Jul 14;7(7):e1002123. doi: 10.1371/journal.ppat.1002123 (PMC3136452; doi:10.1371/journal.ppat.1002123)
Supplement: Figure S3 — Confocal microscopy analysis of control and PML depleted HFs expressing EYFP-PML.I and EYFP-PML.I.Δ7a. A. Control shLuci expressing transduced HFs and derivatives expressing EYFP-PML.I and the PML.I.Δ7a mutant. The upper row shows HF-shLuci control cells stained for endogenous PML and Sp100. The introduced PML.I and PML.I.Δ7a proteins were detected by EYFP autofluorescence and staining for Sp100 (red). B. As A, but in the PML-depleted HF-shPML background. The background cell type, the identity of the detected proteins and the colours used for the merged channels are indicated on each set of panels. Scale bars indicate 5 µm. (PDF) [file ppat.1002123.s003.pdf]

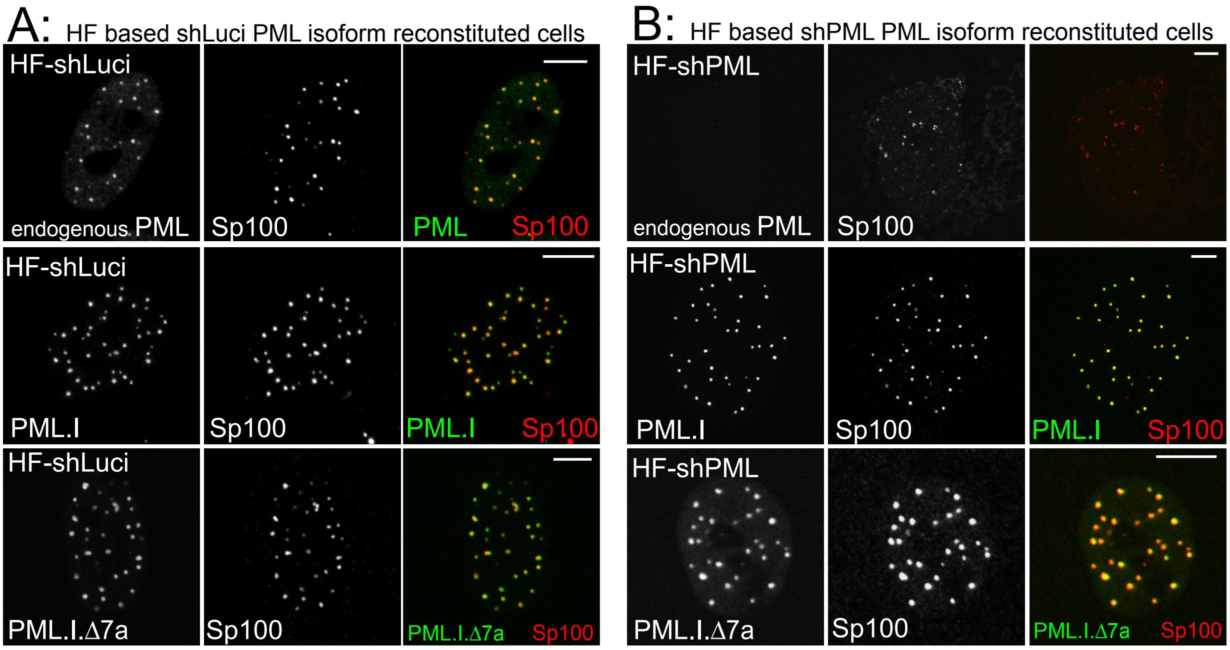
A: HF based shLuci PML isoform reconstituted cells
HF-shLuci
endogenous PML
Sp100
PML
Sp100
HF-shLuci
PML.I
Sp100
PML.I
Sp100
HF-shLuci
PML.I.Δ7a
Sp100
PML.I.Δ7a
Sp100
B: HF based shPML PML isoform reconstituted cells
HF-shPML
endogenous PML
Sp100
HF-shPML
PML.I
Sp100
PML.I
Sp100
HF-shPML
PML.I.Δ7a
Sp100
PML.I.Δ7a
Sp100
